# Non-irradiated area of intraoperative radiotherapy with electron technique: outcomes and pattern of failure in early-stage breast cancer from a single-center, registry study

**DOI:** 10.1007/s12282-024-01624-z

**Published:** 2024-09-10

**Authors:** Tanun Jitwatcharakomol, Jiraporn Setakornnukul, Suebwong Chuthatisith, Adune Ratanawichitrasin, Janjira Petsuksiri, Naponwan Sirima, Kullathorn Thephamongkhol

**Affiliations:** Faculty of Medicine, Siriraj Hospital, Mahidol University, Bangkok, Thailand

**Keywords:** Intraoperative radiotherapy, IORT, Accelerated Partial Breast Irradiation, APBI

## Abstract

**Introduction:**

Intraoperative radiotherapy (IORT) with electrons has revealed to have higher rates of ipsilateral breast tumor recurrence (IBTR) than external beam radiotherapy in updated large-scale, randomized controlled trials in 2021. This study details the oncological outcomes of IORT with electron beams using our strict IORT policies. We have found new and important observations regarding the location of recurrence.

**Methods and materials:**

This is a single institution registry of early-stage breast cancer patients who underwent lumpectomy and electron beam IORT with appropriate cone size. All patients met our pre-excision requirements. The primary endpoint was 5-year IBTR rate, with secondary endpoints being 5-year locoregional failure rate, 5-year distant metastasis rate, 5-year overall survival and, importantly, the failure patterns.

**Results:**

Between January 2011 and December 2022, 124 patients were recruited. The median follow-up was 6.7 years. The 5-year IBTR rate was 1.87% (95% CI 0.47–7.29%), which is much lower than the ELIOT trial and comparable with other accelerated partial breast irradiation (APBI) techniques. The 5-year locoregional failure rate was 3.68% (95% CI 1.40–9.52%), and the 5-year distant metastasis rate was 0.88% (95% CI 0.13–6.12%), while the 5-year overall survival rate was 97.52% (95% CI 92.44–99.19%). Six patients experienced IBTR. All recurrences were in surgical area, occurring superficial to the tumor bed and within 1 cm of the skin dermis. This failure pattern is very unique and might be explained by our hypothesis of the non-irradiated area beneath the skin.

**Conclusions:**

IORT with electron beams with strict patient selection criteria and strict large cone size is still an acceptable treatment for select patients with early-stage breast cancer. However, our new findings support extreme caution in the non-irradiated area beneath the skin around the tumor cavity. Given the constraints of our sample size, these findings should be interpreted cautiously and warrant further investigation in larger, more comprehensive studies.

**Supplementary Information:**

The online version contains supplementary material available at 10.1007/s12282-024-01624-z.

## Introduction

Based on our current understanding of radiobiology, breast cancer cells are classified as late-responding tumors [1], indicating their favorable response to high-dose-per-fraction radiation therapies. In addition, research by Veronesi et al. [2] found that close to 80% of local recurrences are located at the original tumor bed and the scar area. Taken together, these insights paved the way for the concept of accelerated partial breast irradiation. Several landmark trials have reported higher IBTR [3].

Intraoperative radiotherapy (IORT), as one modality of APBI, delivers a concentrated single-dose fraction to the tumor bed during surgery is clearly different from other APBI in terms of no final pathology at the time of patient selection. The American Society for Radiation Oncology (ASTRO) in 2017 [4] has classified patients into three groups: “suitable,” “cautionary,” and “unsuitable”, which is clearly based on the final pathology that could not be entirely revealed before the IORT procedure. Nevertheless, at that time, based on early results of the large two randomized controlled trials (TARGIT-A and ELIOT), which had wider inclusion criteria than the “suitable” group, including tumor size and hormonal status, IORT is advised as an option for those in the suitable category.

However, in recent years, the evidence supporting this ASTRO recommendation have changed. There are a lot of concerns regarding the higher recurrence rate in IORT, compared with other APBI techniques. Several issues have been debated widely regarding both TARGIT-A [5] and ELIOT trials, for example, patient selection criteria, and also the technical issue of IORT in the time of surgery, especially cone size. Importantly, even patients in the “ASTRO suitable group” might not be appropriate for this kind of treatment according to ELIOT long-term results.

The pivotal trial of IORT with electron beams (ELIOT) [6] studied 1305 patients with early-stage breast cancer who underwent breast-conserving surgery. The participants were randomly assigned to one of two treatment groups. One group received 21 Gy ELIOT, while the other group was administered whole-breast EBRT. After a median follow-up of 12.4 years, there were 70 cases of ipsilateral breast tumor recurrence (IBTR) in the ELIOT group, compared to 16 in the EBRT group. These values signified an absolute increase of 54 IBTR cases within the ELIOT group (HR 4.62, 95% CI 2.68–7.95, *P* < 0.0001), surpassing the threshold for non-inferiority. Nonetheless, no significant difference in overall survival was noted between the two groups.

The authors of the ELIOT trial highlighted a significant concern regarding the high IBTR rate. Even within the ASTRO-designated suitable group, the IBTR rate was very high, with a 10-year rate of 6.1% (95% CI 3.6–9.5%) and a 15-year rate of 13.1% (95% CI 8.3–19.1%). Consequently, the primary discussion point of the report on the ELIOT trial was the importance of exercising caution in patient selection, even within the ASTRO suitable groups.

Additionally, a technical issue on the ELIOT trial was raised. A long-term study from Belgium [7] demonstrated a very low IBTR rate when using larger cone sizes for electron IORT; the average cone size used was 5.5 cm for all tumors measuring ≤ 2 cm. This contrasts with the ELIOT trial, which used a smaller average cone size of 4 cm, and only 85% of these cases involved tumors ≤ 2 cm. Additionally, research from the Netherlands [8] indicates a 10-year true IBTR rate of 7.3% in the IORT quadrant for the ASTRO suitable group.

Taken together, these findings suggest that not only patient selection but also technical factors, specifically the narrow cone size, may have contributed to the observed differences in recurrence rates.

The authors of the current research acknowledge the increased recurrence rates reported in the TARGIT and ELIOT trials. Nevertheless, our institution has implemented very stringent patient selection protocols and the Belgium standard regarding cone size. The present study evaluated the outcomes for early-stage breast cancer patients who were selected for IORT with electron beams based on our institution’s very strict pre-excision criteria. The study also determined the interesting failure patterns associated with this treatment method.

## Methods and materials

### Study design

This registry, single-arm study was conducted at Siriraj Hospital in Thailand. Before the research commenced, ethical approval was secured from the Siriraj Ethical Review Board. For this type of study formal consent is not required. We included newly diagnosed female patients with invasive ductal carcinoma of breast cancer that had been pathologically confirmed at any time between the years 2011 and 2022. These patients underwent breast-conserving surgery accompanied by biopsy or dissection of axillary lymph nodes. Complete staging, including evaluations of the bones, liver, and lungs, was conducted either by chest x-ray or by computed tomography (CT) scan of the chest plus, either a CT scan or ultrasonography of the abdomen.

We have a very strict policy for patient selection criteria. To be eligible for IORT with electron beams, patients had to meet the following pre-excision criteria: age ≥ 55 years, tumor size ≤ 2 cm (measured by mammogram or ultrasound), estrogen receptor positivity, no proven of angiolymphatic space invasion and extensive intraductal component, the absence of multicentric tumors, and a sentinel lymph node-negative status confirmed by frozen section analysis at the time of operation. After updated ASTRO reco mmendation of patient selection criteria for APBI in 2016, we have expanded patient selection criteria to include ductal carcinoma in situ (DCIS) and age ≥ 50 years. We excluded patients with metastatic disease (stage M1 per the eighth edition of the American Joint Committee on Cancer staging manual), bilateral breast cancer, or multiple primary malignancies; diagnosed with other cancers in any timeframe whether the disease is control or uncontrol. Patients who had previously undergone radiation therapy to the chest or axillary region were also excluded from the study.

Regarding this patient selection criteria in the IORT setting, which were at the time before the operation, we were aware that there might be additional final pathology that could shift the patient into the cautionary group according to ASTRO 2016 recommendation, for example, surgical margin, which is reasonably not known before operation.

### Intraoperative radiotherapy procedure

IORT was administered using a Mobetron device (Intraop Medical Inc., Santa Clara, CA, USA), delivering 21 Gy to the 90% isodose line encompassing the tumor bed. The radiation oncologist selected electron energies of 6, 9, or 12 MeV and cone sizes ranging from 5 to 7.5 cm according to tumor depth and size measuring intraoperatively. To shield the chest wall, a aluminum-lead disk of the same size as the electron cone was placed atop the pectoralis major muscle. Bolus might be considered to avoid 80% isodose line which cover about 5 mm at the surface for small cone size with low energy (Supplementary Fig. 4).

We have a very strict policy regarding large cone sizes. Our IORT protocol strictly requires at least a 1.5–2 cm margin to cover the tumor bed in every dimension [7]. The appropriate cone size in our center was defined as tumor size plus an additional at least 3 cm. In addition, our surgeons are aware of our protocol to have negative lymph node and adequate surgical margin to be at least 2 mm. In addition, before placing the IORT cone in every patient, we wait for the pathologist to confirm the negative sentinel lymph node from the frozen section.

External beam might be given for patients who have high-risk features such as lymph node positivity confirmed after a full pathological report, positive surgical margins, and extensive intraductal component. The final decision to proceed with external beam radiation after IORT was based on the physician’s and patient’s decisions. If external beam radiation is planned, IORT would be considered as a boost, and whole breast plus regional nodal radiation prescribed at 50 Gy in 25 fractions will be given.

### Follow-up protocol

Patients were assessed with a clinical examination at 3- to 4-month intervals for 2–3 years, followed by biannual examinations. A mammogram plus ultrasound was performed yearly. Additional diagnostic procedures were performed if there were clinical indications of recurrence.

### Statistical analysis

All patients who underwent IORT with electron beams at Faculty of Medicine, Siriraj Hospital, Mahidol University as strict pre-excision patient selection criteria and strict large cone size were included in the study. As mentioned before, although we included with strict suitable pre-excision criteria, some final pathologies could shift some patients to cautionary and unsuitable groups based on the ASTRO guidelines 2016, for example, surgical margin, grade of DCIS, tumor size, and lymph node status (which was negative at the time of frozen section but was positive in final pathology).

The primary endpoint was 5-year IBTR rate, and the secondary endpoints were 5-year locoregional failure rate, 5-year distant metastasis rate, 5-year overall survival, and failure patterns. The “time to event” was calculated from the date of IORT treatment to the occurrence of an event. Our primary outcome is to compare our IBTR rate (95% CI) with ELIOT (4.2%, 95% CI = 2.8–5.9%) and other APBI techniques from randomized controlled trials [9–11] reported in the literature (point estimate around 0.5–2.3%, 95% CI = 0.2–3.2%). The Kaplan–Meier method with log-rank tests was also employed to analyze IBTR, locoregional control, distant metastasis-free survival, and overall survival rates. The statistical analysis was conducted using Stata Statistical Software, release 18 (StataCorp LLC, College Station, TX, USA). Failure patterns were newly determined through physical examination and diagnostic imaging newly reviewed by certified diagnostic radiologist. Importantly, we have also tried to map the recurrence area with the surgical cavity, dose distribution of electrons, and surgical area to explain the reason for each recurrence.

## Results

Between January 2011 and December 2022, 282 patients with newly diagnosed early-stage breast cancer were meet our pre-excision criteria for IORT. In these number, 152 patients underwent breast conservative surgery and IORT with electron beams. Of these, 28 were excluded due to bilateral breast cancer (14 patients) or a second primary malignancy (14 patients), leaving 124 patients for analysis.

Patient demographics and tumor characteristics are detailed in Table 1. The mean patient age was 65 years. Most patients had cancer in the left breast (52.4%) located in the upper outer quadrant (46%). The mean tumor size was 1.21 cm, predominantly invasive ductal carcinoma (91.1%) and primarily histological grade 2 (58.9%). Single foci were present in 96% of patients. Luminal B was the most frequent intrinsic subtype, found in 46.8% of cases. Systemic hormonal therapy was administered to 119 patients (96%).

**Table 1:** Patient characteristics

Total (*N* = 124)	
Age (year)	
50–54	4 (3.2%)
55–60	32 (25.8%)
> 60	88 (71.0%)
Laterality	
Right	59 (47.6%)
Left	65 (52.4%)
In-breast location	
Upper inner	33 (26.6%)
Upper outer	57 (46.0%)
Lower inner	9 (7.3%)
Lower outer	14 (11.3%)
Center	11 (8.9%)
Tumor size (cm), mean (SD)	1.21 (0.59)
Tumor stage	
T1a	14 (11.3%)
T1b	42 (33.9%)
T1c	60 (48.3%)
T2	8 (6.5%)
Grade	
Grade 1	36 (29.0%)
Grade 2	73 (58.9%)
Grade 3	15 (12.1%)
Histology	
Invasive ductal carcinoma	113 (91.1%)
Other histology*	5 (4.0%)
Pure DCIS	6 (4.8%)
Focality	
Single	122 (98.4%)
Multiple	2 (1.6%)
LVSI	
Negative	117 (94.3%)
Positive	7 (5.6%)
PNI	
Negative	14 (11.3%)
Positive	11 (8.9%)
N/A	99 (79.8%)
ER status	
Positive	100 (100.0%)
PR status	
Negative	8 (6.5%)
Positive	116 (93.5%)
Her-2 status	
Negative	102 (82.3%)
Equivocal	9 (7.3%)
Positive	6 (4.8%)
N/A	7 (5.6%)
Ki-67 (%), mean (SD)	20.55 (11.68)
Intrinsic subtype	
Luminal A	25 (20.2%)
Luminal B	58 (46.8%)
Luminal (Non-classified A/B)	41 (33.1%)

Data are presented as *n* (%) unless otherwise indicated

Abbreviations:* LVSI* lymphovascular space invasion,* PNI* perineural space invasion,* ER* Estrogen receptor,* PR* Progesterone Receptor,* Her-2* Human Epidermal Growth Factor Receptor 2,* N/A* not applicable

^*^Other histology included solid papillary carcinoma and encapsulated papillary carcinoma

Systemic and IORT treatment details were noted (Table 2). The mean tumor cavity depth was 2.92 cm, while the mean depth to the chest wall was 3.96 cm. The most frequently used electron energy source was 12 MeV, with cone sizes of 6 cm and 7 cm. The mean difference between tumor size and cone size was 5.05 cm. Most patients did not use a bolus (75%), but almost all patients used a shield (92.7%). The mean tumor isodose achieved was 92.69%. Only three patients (2.4%) received EBRT following IORT due to lymph node positive (2 patients) and close DCIS margin (1 patient).

**Table 2:** Treatment characteristics

Total (*N* = 124)	
Systemic treatment	
No	2 (1.6%)
Yes	119 (96.0%)
N/A	3 (2.4%)
Systemic regimen	
Hormone therapy alone	103 (85.7%)
Chemotherapy + Hormone	17 (14.3%)
Chemotherapy regimen	
Adriamycin + Cyclophospohamide	11 (64.7%)
Docetaxel + Cyclophospohamide	6 (35.3%)
Hormonal therapy regimen	
Tamoxifen	48 (40.3%)
Aromatase inhibitor	35 (29.4%)
Tamoxifen + Aromatase inhibitor	36 (30.3%)
Tumor Cavity Depth* (cm)	
≤ 2	19 (15.3%)
> 2	105 (84.7%)
Energy (MeV)	
6	19 (15.3%)
9	35 (28.2%)
12	70 (56.5%)
Cone size (cm)	
5	8 (6.3%)
5.5	15 (12.1%)
6	46 (37.1%)
6.5	17 (13.7%)
7	37 (29.8%)
7.5	1 (0.8%)
Cone size and tumor size difference (cm),mean (SD)	5.05 (0.75)
Bolus	
No	93 (75.0%)
Yes	31 (25.0%)
Tumor Isodose (%), mean (SD)	92.69 (6.93)
Chest wall depth (cm), mean (SD)	3.96 (0.95)
Chest wall Isodose (%), mean (SD)	61.07 (24.88)
Shield	
No	9 (7.3%)
Yes	115 (92.7%)
Chest wall dose (Gy),mean (SD)	1.65 (1.34)
Received external beam treatment	
No	121 (97.6%)
Yes	3 (2.4%)

Data are presented as *n* (%) unless otherwise indicated

*N/A* not applicable

^*^Tumor cavity depth is measured from skin to deepest part of tumor cavity in the operation room

Following post-excision final pathological analysis, the 124 patients were categorized into three groups according to the ASTRO (2017) [4] categories (Supplementary Table 1). Seventy-four patients (59.68%) were classified as suitable, 45 patients (36.29%) as cautionary, and five patients (4.03%) as unsuitable. We were also categorized the patients into four groups according to the recently updated ASTRO (2024) [12] (Supplementary Table 3). Most of patient in this study was recommended for partial breast irradiation (62.1%), whereas only 4% was not recommended. Details of patterns of failure are available in Supplementary Table 4. However due to lack of Her-2 status in 12.9% of our patients, we could not classify these patient into the appropriate group according to ASTRO (2024) definition. Thus, the analysis will be provided base on the ASTRO (2017) categories.

**Fig. 1:**
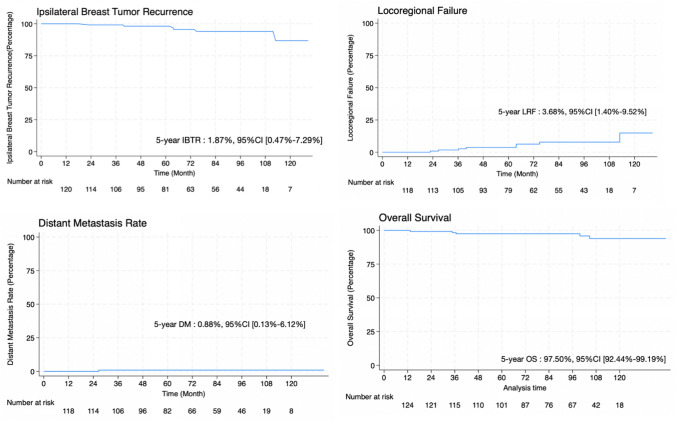
Ipsilateral breast tumor recurrence (IBTR), locoregional failure (LRF), distant metastasis rate (DM), overall survival (OS)

In this study, the median follow-up duration was 6.7 years. Our main outcome, the 5-year IBTR was 1.87% (95% CI 0.47–7.29%), The 5-year locoregional failure rate was 3.68% (95% CI 1.40–9.52%) (Fig. 1). There were six instances of IBTR (Supplementary Table 2), with half occurring in the suitable category (1.54%, 95% CI 0.22–10.42%) and the other half in the cautionary category (2.5%, 95% CI 0.36–16.45%). No events were observed in the unsuitable subgroup, resulting in a 5-year local control rate of 100% (Supplementary Fig. 1). All six recurrences were in surgical area but outside the irradiated area, primarily 1–2 cm from the initial tumor bed. Remarkably, we have found all recurrences were at superficial location, at a depth of less than 1 cm from the skin dermis to superficial edge of recurrent tumor (Table 3).

**Table 3:** Ipsilateral breast tumor recurrence details

IBTR Patient	Patient group	Initial tumor location	Recurrence Tumor location	Tumor size(cm)	Invasive surgical margin (mm)	Extensive intraductal component	Electron Energy (MeV)	Applicator size (cm)	Tumor cavity depth at time of IORT (cm)	Distant from superficial edge of recurrent tumor to skin dermis (cm)	Distant from nearest clip to recurrent tumor in CC view (cm)	Distant from nearest clip to recurrent tumor in MLO view (cm)
1	Cautionary	UIQ	UIQ	1.1	10	Negative	12	7	4	0.84	0.10	0
2	Cautionary	LOQ	LOQ	1.7	3	Negative	9	6.5	2.6	0.24	0.77	N/A
3	Cautionary	UIQ	UIQ	0.8	2	Negative	9	6	2.5	0.94	N/A	1.45
4	Suitable	UOQ	UOQ	1.5	5	Negative	12	5.5	3.8	Invade	1.60	1.72
5	Suitable	UIQ	UIQ	1.7	4	Negative	9	6	2.7	Invade	2.29	2.56
6	Suitable	UIQ	UIQ	1	7	Negative	12	6	4	0.48	N/A	3.45

Abbreviations: *IBTR* ipsilateral breast tumor recurrence, *LUQ* lower outer quadrant, *UIQ* upper inner quadrant, *UOQ* upper outer quadrant, *N/A* not applicable due to breast density, *CC view* craniocaudal view, *MLO view* mediolateral oblique view

The 5-year distant metastasis rate was 0.88% (95% CI 0.13–6.12%). and the 5-year overall survival rate was 97.52% (95% CI 92.44–99.19%).

Univariable and multivariable analyses of IBTR were conducted (Table 4). Upper inner in-breast location and LVSI were identified as significant risk factors in both analyses.

**Table 4:** Univariate and multivariate analyses of ipsilateral breast Tumor Recurrence

Variable	Event/total (%)	Univariate		Multivariate	
		HR [95% CI]	*P*	HR [95% CI]	*P*
In-breast location					
Non-upper inner	3/92 (3.26%)	Ref			
Upper inner	3/32 (9.38%)	5.70 [1.04–31.21]	0.045	17.10 [1.79–163.11]	0.014
LVSI					
No	5/117 (4.27%)	Ref			
Yes	1/7 (14.3%)	7.24 [0.80–65.70]	0.078	91.52 [1.83–4573.02]	0.024
Tumor cavity depth	6/124 (4.84%)	2.11 [0.65–6.83]	0.214	4.37 [0.89–21.45]	0.070
Age	6/124 (4.84%)	1.00 [0.88–1.13]	0.987	0.93 [0.75–1.15]	0.505
Site					
Right	3/59 (5.08%)	Ref			
Left	3/65 (4.62%)	1.10 [0.22–5.47]	0.909	1.37 [0.15–12.20]	0.780
Tumor size	6/124 (4.84%)	1.35 [0.34–5.30]	0.665	5.47 [0.57–52.68]	0.142
Patient group					
Suitable	3/74 (4.05%)	Ref			
Cautionary/unsuitable	3/50 (6%)	1.66 [0.33–8.36]	0.540	0.30 [0.02–3.80]	0.353
Grade					
1	2/36 (5.56%)	Ref			
2	3/73 (4.11%)	0.95 [0.16–5.71]	0.957	0.75 [0.09–6.11]	0.789
3	1/15 (6.67%)	1.67 [0.15–18.62]	0.677	0.42 [0.02–11.21]	0.604
Intrinsic subtype					
Luminal A/B	3/83 (3.61%)	Ref			
Non-classified	3/41 (7.32%)	1.45 [0.27–7.76]	0.665	1.60 [0.13–19.73]	0.716

Abbreviations: *LVSI* lymphovascular space invasion, *PNI* perineural space invasion, *N/A* not applicable

Toxicity profiles [13] and cosmetic outcomes [14] after IORT were reports at Supplement Tables 5 and 6. Only 4.8% G2 acute dermatitis was observed, without any heart and lung toxicity. Excellent cosmetic outcomes were observed, with important aspects such as breast shape distortion, skin color changes, and tissue induration showing notable improvement. These factors demonstrated a trend towards enhancement from the initial follow-up to the 1–2-year assessment period.

## Discussion

With a median follow-up of 6.7 years, our study showed a 5-year IBTR rate of 1.87% (95% CI 0.47–7.29%), which is comparable with the 5-year IBTR rate of the Belgian study and other APBI techniques (0.5–2.7%) [7, 9–11] and lower than that of ELIOT trial and Netherland trial, which is 4.2% and 10.6%, respectively.

Our IORT technique mirrored that of the ELIOT trial, which involved administering a single 21 Gy dose of electron beam radiotherapy intraoperatively after tumor excision. The difference may be due to our use of more favorable patient selection criteria, such as the exclusion of lymph node-positive and triple-negative patients, a greater proportion of estrogen receptor/progesterone receptor-positive patients, and a high rate of adjuvant systemic therapy.

Another contributing factor to our lower 5-year IBTR rate could be the use of larger cone sizes of 6 and 7 cm in 67.5% of our patients, covering a mean tumor size of 1.21 cm, and the mean difference between cone size and tumor size is 5.05 cm. This approach is in line with a Belgian study [7] that used an average cone size of 5.5 cm for tumors ≤ 2 cm in unifocal early-stage breast cancer, resulting in a 5-year IBTR rate of 2.7%. The reduced 5-year IBTR in our study may be attributable to our use of appropriate applicator cone sizes, paralleling the approach in the Belgian study.

Subgroup analysis in our study was performed based on the “ASTRO” definition. 5-year IBTR of 1.54% (95% CI 0.22–10.42%) was observed in the “suitable” group of patients which was lower than the result from a suitable subgroup in the ELIOT trial (2.0%, 95% CI 0.8–4.4%). In addition, we did not find any IBTR events in the unsuitable subgroup, however, it might be because of the extremely low number of patients in this subgroup.

Univariable and multivariable analyses were performed. LVSI was confirmed as a significant negative prognostic factor. We highly recommend excluding these groups of patients from IORT treatment. If the post-excision pathology shows a positive LVSI after IORT was giving, consideration might be given to adding external beam radiotherapy.

Another negative factor was the initial tumor location at the inner upper quadrant. In our study, four local recurrent patients were in the inner upper quadrant. The inner upper quadrant seems to have less breast tissue compared to other quadrants. Consequently, when performing IORT with electron beams, there can be challenges in aligning and stitching together the two breast-tissue flaps due to the tightness of the breast. This tightness could lead to some areas being inadequately encompassed by the electron beam, necessitating broader beam coverage.

Given the rarity of failures in the suitable and cautionary groups, we conducted a thorough review of the patients who experienced IBTR. All six patients with IBTR experienced in-field failures situated superficially, less than 1 cm from the skin dermis to superficial edge of recurrent tumor (Supplementary Fig. 2). To explain these findings, we developed the following hypothesis based on the surgical procedure described in the ELIOT trial.

The surgical technique in the ELIOT trial [15] employed a quadrantectomy with 1–2 cm clear margins. Following quadrantectomy, the breast tissue was typically reapproximated to close the surgical wound. During the IORT procedure, the separated anterior and posterior breast tissue flaps were temporarily stitched together before applying a vertically oriented radiation beam.

**Fig. 2:**
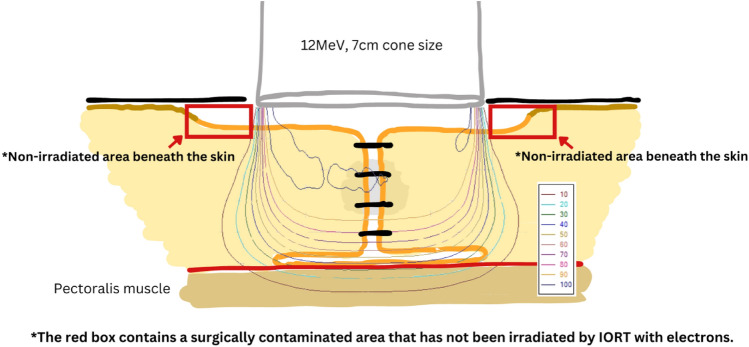
Surgical procedure and electron beam profile during IORT

By employing this IORT method (Fig. 2), the breast tissue beneath the skin around the tumor cavity was detached to enable pulling and stitching it closely together after the tumor was removed. This additional step increases the likelihood of contamination area occurring during surgery in the area beneath the skin. Although the breast tissue under the skin was pulled together to receive the IORT treatment, another contamination area, the area beneath the skin, was intentionally not pulled, as we aim to keep the skin outside the IORT field. Despite the low number of IBTR events in this study, our observations suggest that the non-irradiated area could be a potential area of interest in this radiation method. While these findings are limited by the small sample size, they may indicate an aspect worth exploring in future research.

Additionally, the most common settings in this study were 12 MeV energy with a 6- or 7-cm cone size. Based on the electron beam profiles (Fig. 2), the beam entry at the surface is sharply delineated by the applicator, creating a narrow penumbra. However, deeper within the tissue, the penumbra widens before the dose falls off sharply with depth. A wider penumbra implies that there is more coverage in the deeper areas compared to the superficial areas. The extremely narrow penumbra at superficial areas cannot cover the contamination area underneath the skin, as previously mentioned.

Our explanations might clarify why the recurrences in our study occurred near the tumor cavity and situated superficially, less than 1 cm from the skin dermis. These limitations in the surgical procedure and the characteristics of the electron beams might account for the predominance of superficial recurrences over deep tissue failures.

This finding is a novel aspect of our research. This hypothesis might explain why IORT with electron beams showed a higher IBTR rate than other accelerated partial breast irradiation techniques, such as EBRT, which do not demonstrate an increased IBTR rate [9, 10, 16]. Accelerated partial breast irradiation using EBRT requires covering all surgical distortion and cavities with an isotropic margin of 1 or 1.5 cm. This approach reduces the likelihood of missing the target area, a potential issue with IORT with electron beams in our study.

Our preliminary results point to a possibly important consideration in this radiation technique: the non-irradiated region. However, we acknowledge that the small number of IBTR cases limits the generalizability of these observations. This warrants further investigation through larger studies or comparisons with other methods to better understand its significance and potential implications for improving the technique.

According to the reasons mentioned earlier, our data suggested that IORT with electrons should be employed with extreme caution. It is highly recommended to incorporate an additional step in the surgery, such as changing the operation equipment before detaching the breast tissue beneath the skin or detaching the breast tissue beneath the skin as little as possible, to minimize the risk of increasing contamination in the non-irradiated area beneath the skin.

Our strength in this study was the strict policy and cone size technique. We believe that our finding of characteristics of failure has never been discussed and never been reported elsewhere. These findings could be the explanations for the high rate of IBTR in IORT with electron beams which has been argued for decades.

There are several limitations to consider in this study. First, it was a retrospective study, so we could not prospectively collect and measure certain important information. Second, this study had a relatively short-term follow-up of 6.7 years, which may limit our ability to fully assess the long-term outcomes and potential complications associated with IORT using electron beams. Although our study reported a low rate of IBTR, however, long-term follow-up is needed.

According to the recently published, ASTRO Clinical Practice Guideline for Partial Breast Irradiation [12], electron IORT is not recommended. However, supported by our data with a 5-year IBTR rate of 1.87% and a 5-year overall survival rate was 97.52% with excellent cosmetic outcomes. IORT with electron beams with strict patient selection criteria and large cone size may be a viable alternative treatment option for early-stage breast cancer.

## Conclusions

IORT with electron beams with strict patient selection criteria and strict large cone size is still an acceptable treatment for select patients with early-stage breast cancer. However, our new findings support extreme caution in the non-irradiated area beneath the skin around the tumor cavity. Given the constraints of our sample size, these findings should be interpreted cautiously and warrant further investigation in larger, more comprehensive studies.

## Supplementary Information

Below is the link to the electronic supplementary material.

Supplementary file1 (DOCX 2299 KB)

## Data Availability

Research data are stored in an institutional repository and will be shared upon request to the corresponding author.
