# Supplementary material for: Non-irradiated area of intraoperative radiotherapy with electron technique: outcomes and pattern of failure in early-stage breast cancer from a single-center, registry study
Source: Breast Cancer. 2024 Sep 10;31(6):1092–100. doi: 10.1007/s12282-024-01624-z (PMC11489276; doi:10.1007/s12282-024-01624-z)
Supplement: Supplementary file 1 — Supplementary file1 (DOCX 2299 KB) [file 12282_2024_1624_MOESM1_ESM.docx]

**Supplementary**

**Figure 1**: Local Control Based on Patient Classification Subgroups (ASTRO 2017)

**Figure 2**: Failure Pattern

**Figure 3**: Tumor Cavity Depth and Local Recurrence Probability Relationship

**Figure 4**: Isodose line in different cone sizes and electron energies

**Table 1**: Patient Classification per ASTRO 2017

**Table 2**: First Failure Site per ASTRO 2017

**Table 3**: Patient Classification per ASTRO 2024

**Table 4**: First Failure Site per ASTRO 2024

**Table 5**: Toxicity profiles (CTCAE V5.0)

**Table 6**: Cosmetic profiles

**Figure 1**: Ipsilateral Breast Tumor Recurrence Based on Patient Classification Subgroups (ASTRO2017)


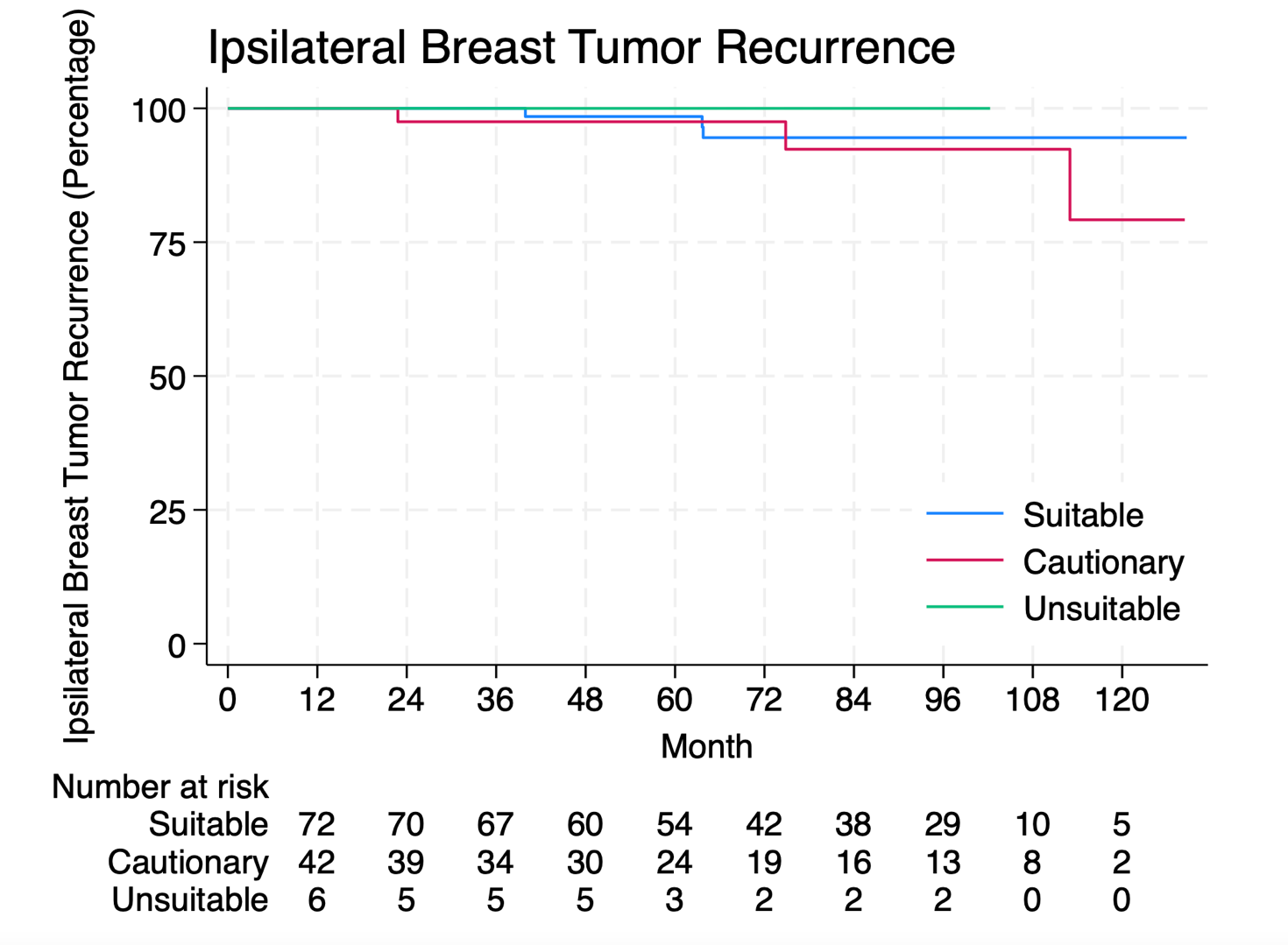


**Figure 2**: Failure Pattern

**
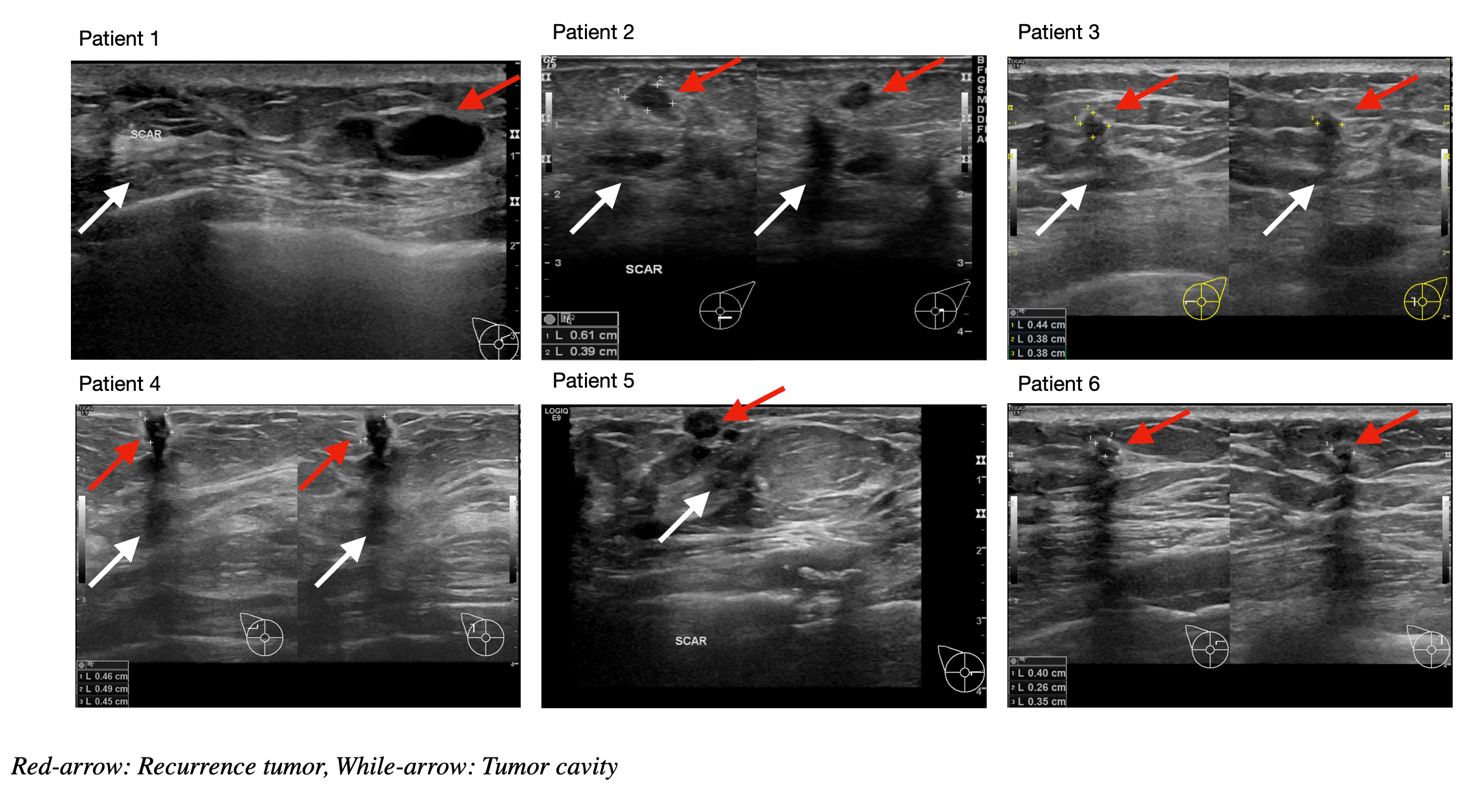
**

**Figure 3**: Tumor cavity depth and ipsilateral breast tumor recurrent probability relationship


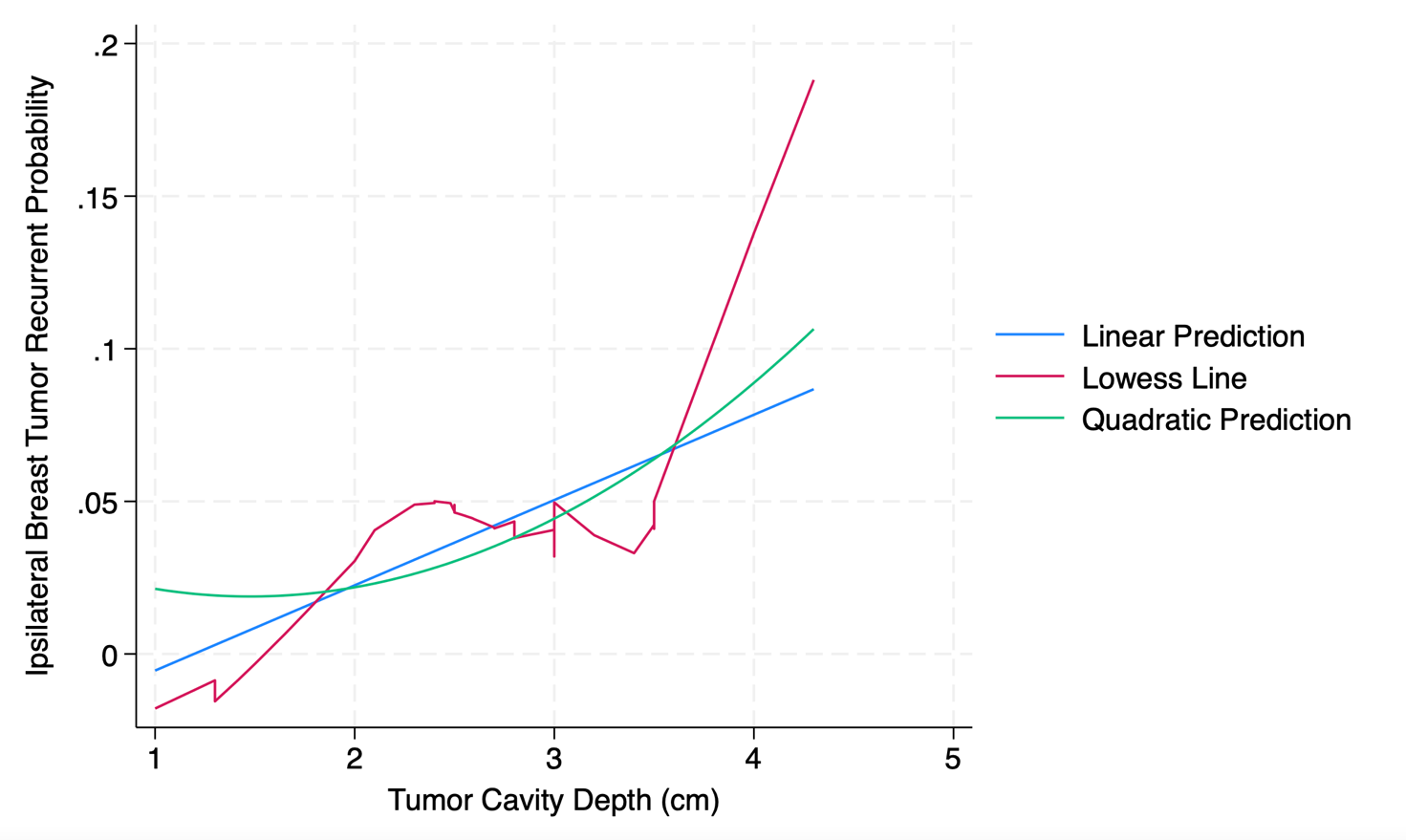


**Figure 4:** Isodose line in different cone sizes and electron energies

**
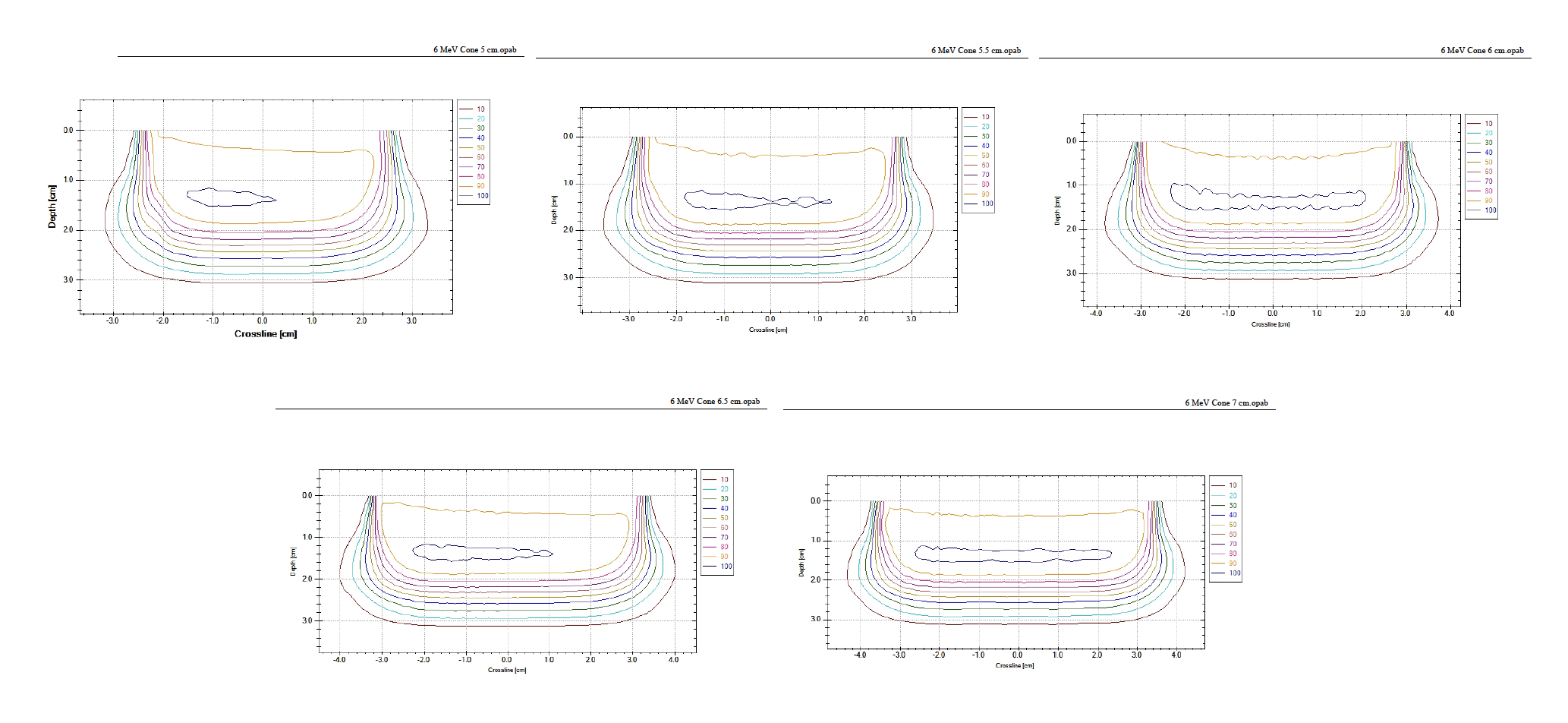
**

**
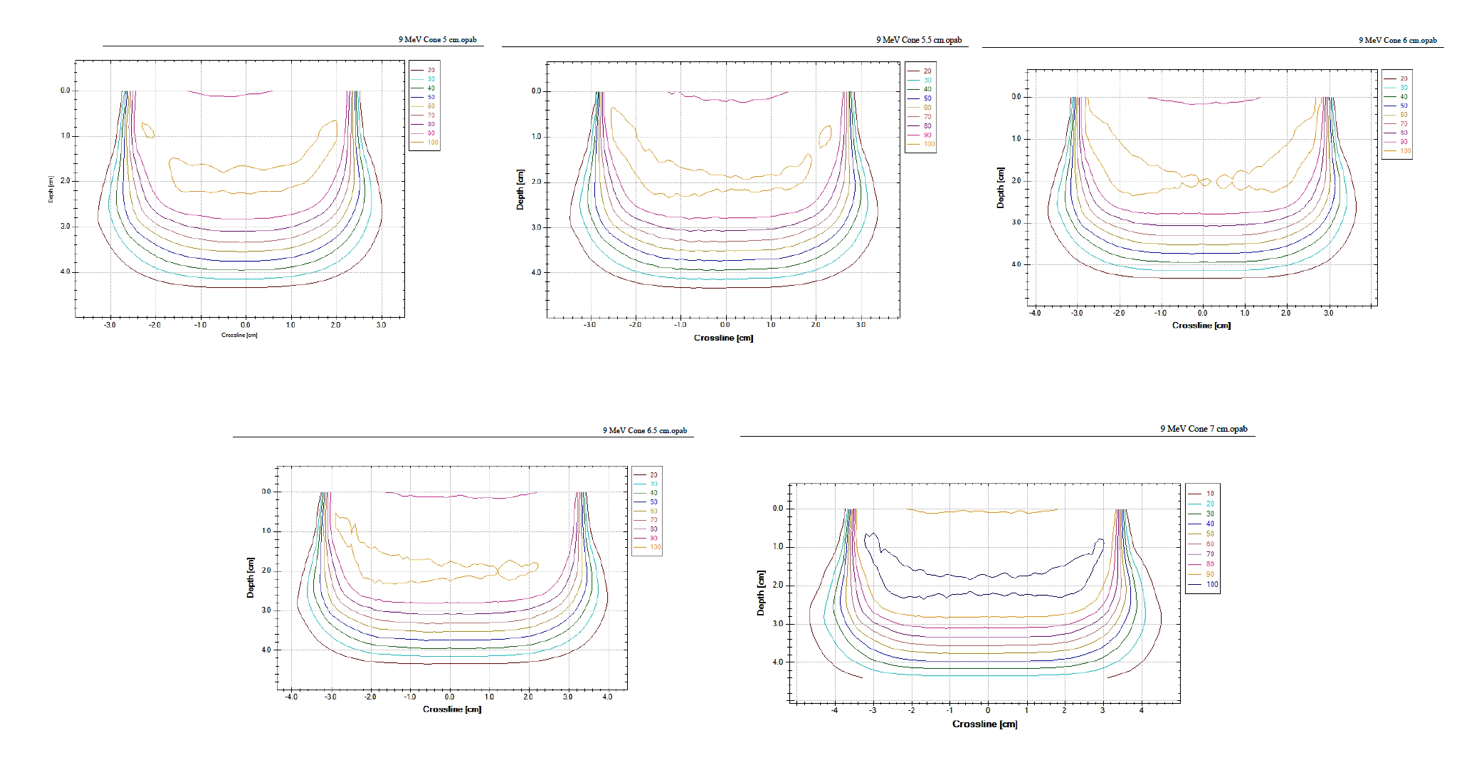
**

**
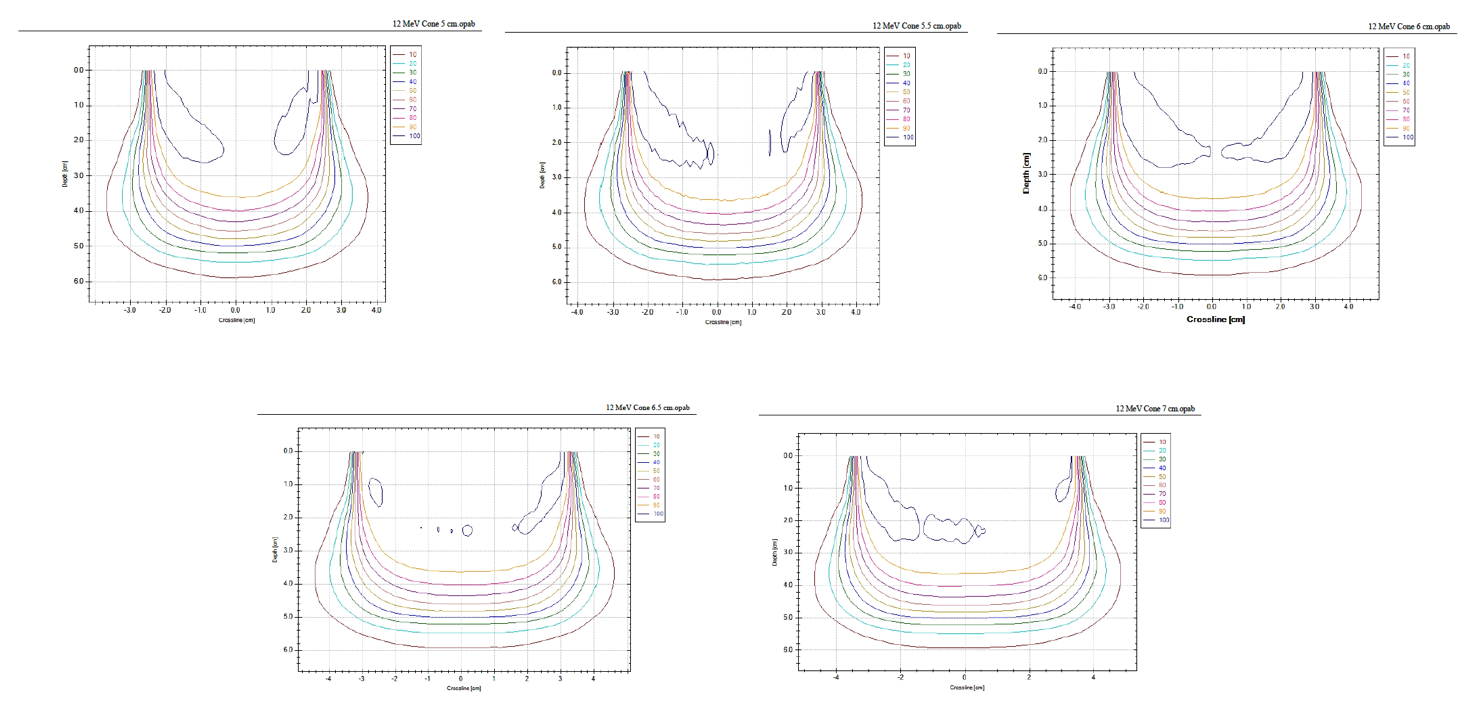
**

**Table 1**: Patient Classification per ASTRO (2017)

| Patient Classification | Number (%) |
| --- | --- |
| **Suitable** | **74 (59.68%)** |
| **Cautionary** | **45 (36.29%)** |
| IDC with close margin (<2mm) | 3 |
| DCIS with close margin (<3mm) | 11 |
| IDC and DCIS with close margin | 8 |
| High grade DCIS | 9 |
| Focal LVSI positive | 7 |
| Tumor size 2-3 cm | 7 |
| **Unsuitable** | **5 (4.03%)** |
| Multifoci tumor | 2 |
| Size > 3 cm | 1 |
| Lymph node positive | 2 |
| **Total** | **124 (100%)** |

*Abbreviations: IDC = invasive ductal carcinoma; DCSI = ductal carcinoma in situ; LVSI = lymphovascular space invasion*

*Data are presented as n (%) unless otherwise indicated.*

**Table 2**: First Failure Site per ASTRO (2017)

| **First failure**  **n (% in group)** | Patient group | | |
| --- | --- | --- | --- |
|  | Suitable | Cautionary | Unsuitable |
| **Local** | 3 (4%) | 3 (6.7%) | 0 (0%) |
| **Regional** | 2 (2.7%) | 0 (0%) | 0 (0%) |
| **Distant** | 0 (0%) | 0 (0%) | 0 (0%) |
| **Total** | **5 (6.7%) / 74** | **3 (6.7%) / 45** | **0 (0%) / 5** |

*Data are presented as n (%) unless otherwise indicated.*

**Table 3**: Patient Classification According ASTRO (2024)

| Patient Classification | Number (%) |
| --- | --- |
| **Recommended** | **77 (62.1%)** |
| **Conditionally recommended** | **14 (11.3%)** |
| **IDC grade 3** | **6** |
| **DCIS grade 3** | **4** |
| **IDC + DCIS both grade 3** | **1** |
| **Tumor Size 2-3 cm** | **2** |
| **IDC grade 3 + Tumor size 2-3cm** | **1** |
| **Conditionally not recommended** | **13 (10.5%)** |
| **LVSI positive** | **7^a^** |
| **Her-2 positive not receiving Herceptin** | **6** |
| **Not recommended** | **5 (4%)** |
| **Multifocal tumor** | **2** |
| **Size > 3cm** | **1** |
| **Lymph node positive** | **2** |
| **N/A*** | **15 (12.1%)** |
| **Her-2 status: equivocal** | **8** |
| **Her-2 status: N/A** | **7** |
| **Total** | **124 (100%)** |

** Cannot classified due to lack of Her-2 Data*

*^a^ one patient of Her-2 status equivocal was LVSI positive so we are able to classify the patient into conditionally not recommended.*

*Abbreviations: IDC = invasive ductal carcinoma; DCSI = ductal carcinoma in situ; LVSI = lymphovascular space invasion; N/A = not applicable*

*Data are presented as n (%) unless otherwise indicated.*

**Table 4**: First Failure Site per ASTRO (2024)

| **First failure**  **n (% in group)** | **Patient group** | | | | |
| --- | --- | --- | --- | --- | --- |
|  | **Recommended** | **Conditionally recommended** | **Conditionally not recommended** | **Not recommended** | **N/A*** |
| **Local** | 3 (3.9%) | 1 (7.1%) ^a^ | 1 (7.7%) ^b^ | 0 (0%) | 1 (7.1%) ^c^ |
| **Regional** | 1 (1.3%) | 0 (0%) | 1 (7.7%) ^d^ | 0 (0%) | 0 (0%) |
| **Distant** | 0 (0%) | 0 (0%) | 0 (0%) | 0 (0%) | 0 (0%) |
| **Total** | **4(5.2%)/77** | **1(7.1%)/14** | **2(15.4%)/ 13** | **0(0%)/ 5** | **1(7.1%)/ 14** |

** Cannot classified due to lack of Her-2 Data*

*^a^ this patient has high grade invasive ductal carcinoma.*

*^b^ this patient has focal lymphovascular space invasion.*

*^c^ this patient has Her-2 status equivocal not receiving Herceptin.*

*^d^ this patient has Her-2 status positive not receiving Herceptin.*

*Abbreviations: N/A = not applicable*

*Data are presented as n (%) unless otherwise indicated.*

**Table 5**: Toxicity profiles (CTCAE V5.0)

| **Acute Toxicity (n=124)** | **G0** | **G1** | **G2** | **G3** | **G4** | **N/A** |
| --- | --- | --- | --- | --- | --- | --- |
| Dermatitis radiation | 91 (73.5%) | 24 (19.3%) | 6 (4.8%) | 0 (0%) | 0 (0%) | 3 (2.4%) |
| Pneumonitis | 121 (97.6%) | 0 (0%) | 0 (0%) | 0 (0%) | 0 (0%) | 3 (2.4%) |
| **Late Toxicity (n=124)** | **G0** | **G1** | **G2** | **G3** | **G4** | **N/A** |
| Heart failure | 119 (96%) | 0 (0%) | 0 (0%) | 0 (0%) | 0 (0%) | 5 (4%) |
| Pulmonary fibrosis | 119 (96%) | 0 (0%) | 0 (0%) | 0 (0%) | 0 (0%) | 5 (4%) |
| **Total** |  |  |  |  |  |  |

** Skin infection required intervention were included in G3 radiation dermatitis.*

*Data are presented as n (%) unless otherwise indicated.*

**Table 6**: Cosmetic profiles

|  | **Cosmetic outcomes at 1^st^ follow-up (n=47)** | | | **Cosmetic outcomes at 1-2 year (n=39)** | | | |
| --- | --- | --- | --- | --- | --- | --- | --- |
|  | **None** | **Mild** | **Moderate** | **None** | **Mild** | **Moderate** |  |
| **Shape distortion** | 21 (44.7%) | 25 (53.2%) | 1 (2.1%) | 29 (74.4%) | 10 (25.6%) | 0 (0%) |  |
| **Skin color change** | 40 (85.1%) | 7 (14.9%) | 0 (0%) | 38 (97.4%) | 1 (2.6%) | 0 (0%) |  |
| **Tissue Induration** | 8 (17%) | 33 (70.2%) | 6 (12.8%) | 20 (51.3%) | 19 (48.7%) | 0 (0%) |  |

*Data are presented as n (%) unless otherwise indicated.*

*No cosmetic outcome “severe” was observed.*
